# Performance of the Cognitive Performance Scale of the Resident Assessment Instrument (interRAI) for Detecting Dementia amongst Older Adults in the Community

**DOI:** 10.3390/ijerph18136708

**Published:** 2021-06-22

**Authors:** Susan Gee, Matthew Croucher, Gary Cheung

**Affiliations:** 1 Psychiatry of Old Age Academic Unit, Burwood Hospital, Private Bag 4708, Christchurch 8140, New Zealand; matthew.croucher@cdhb.health.nz; 2 Auckland Mail Centre, Department of Psychological Medicine, The University of Auckland, Private Bag 92019, Auckland 1142, New Zealand; g.cheung@auckland.ac.nz

**Keywords:** interrail, dementia, cognitive assessment, diagnostic accuracy, validation studies, geriatric assessment

## Abstract

The Cognitive Performance Scale (CPS) in the widely used interRAI suite of instruments is of interest to clinicians and policy makers as a potential screening mechanism for detecting dementia. However, there has been little evaluation of the CPS in home care settings. This retrospective diagnostic study included 134 older adults (age ≥ 65) who were discharged from two acute psychogeriatric inpatient units or assessed in two memory clinics. The reference test was a diagnosis of clinical dementia, and the index test was interRAI CPS measured within 90 days of discharge. The overall accuracy of the CPS was good, with an area under the Receiver Operating Characteristic curve of 0.82 (95% CI = 0.75–0.89). The optimal cut point was 1/2, coinciding with the recommended cut point, with good sensitivity (0.90, 95% CI = 0.81–0.96) but poor specificity (0.60, 95% CI = 0.46–0.72). Positive predictive value improved from 0.72 (95% CI = 0.66–0.78) to 0.89 (95% CI = 0.75–0.96) when using a cut point of 2/3 instead of 1/2. If the results of the present study are replicated with more generalisable interRAI samples, older adults with a CPS of 3 or above, but without a formal diagnosis of dementia, should be referred for further cognitive assessment.

## 1. Introduction

Dementia has been identified by the World Health Organisation as a global public health priority [1]. While it is estimated that almost 9.9 million people develop dementia annually, only a minority of these people will receive a diagnosis of dementia at an early stage in the disease, even in developed countries [2]. The timely detection of cognitive impairment is a cornerstone of public health initiatives to reduce the secondary impact of dementia, such as reducing care-partner stress by providing early support [3]. It can also help identify reversible causes such as depression, vitamin deficiencies, or delirium. Where a dementia diagnosis is confirmed, the quality of care can be improved by providing access to information, support services, and cognition-maintaining interventions [4]. There are major challenges, however, to be addressed to encourage the earlier recognition of cognitive impairment and integration of services in order to open up access to community support, care-giver support, and health and social services [5]. Robust routine assessment of cognitive function and accurate screening in selected high-risk populations may offer a way to help address unmet needs and strengthen decision making for people with dementia [6].

In New Zealand, the Cognitive Performance Scale (CPS), which can be derived from some routinely collected interRAI (international Resident Assessment Instrument) measures, has generated interest as a potential aid for the process of identifying people living with undiagnosed dementia in the community. However, significant questions about the suitability of the CPS for this task also exist. 

New Zealand was the first country to mandate use of selected tools from the International Resident Assessment Instrument or interRAI suite. Mandated tools include the Contact, Home Care, Community Health, Acute Care, Long-Term Care Facilities, and Palliative Care assessments, with each one being used in different clinical settings, including for all older adults who are being considered for access to publicly funded community services or residential care [7]. The interRAI aims to provide a comprehensive clinical assessment of medical, rehabilitation, and support needs and abilities [8,9]. This information can support care planning, resource allocation, quality measurement and outcome evaluation [8]. Client Assessment Protocols (CAPs) are core outputs generated from the interRAI assessment that are used to identify specific clinical conditions or situations and inform care plans. Various ancillary clinical measures have also been developed within the interRAI instrument, including the CPS.

**Figure 1:**
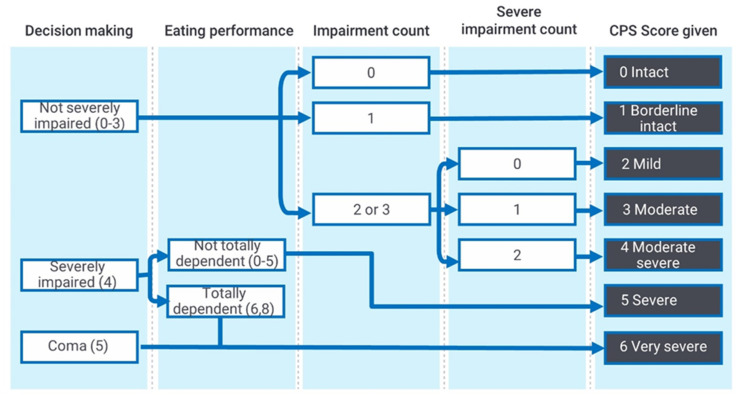
Cognitive Performance Scale (CPS) algorithm (developed by Morris et al. [9]). Figure Note: Impairment count is a count of the following: Decision Making: Not Independent (rating of 1–3); Understood: Not Independent (1–4); Short-Term Memory: Not OK (1). Severe Impairment Count is a count of the following: Decision Making: Moderate Impairment (3); Understood: Sometimes/Never (3–4).

The CPS was developed by Morris and colleagues [9] by searching for a hierarchical algorithm of items in the RAI that would best predict scores on the Mini Mental State Examination (MMSE) [10] and the Test for Severe Impairment [11] in a sample of 136 aged care facility residents. As can be seen in Figure 1, the CPS scale uses individual items on decision making/coma and eating performance as well as a count of the number of impairments and of severe impairments to create a hierarchical algorithmthat provides scores from 0 (no cognitive impairment) to 6 (severe cognitive impairment). A CPS score of ≥2 indicates the probable presence of clinically significant cognitive impairment. The CPS is generated from a selection of items based on reports of clinical problems gathered in an interview format by registered health professionals. It does not involve independent testing of cognitive performance. Researchers have noted that the rating decisions made in this format are not immune to influence from factors other than cognition, such as the time of day the data were collected and the age of the person being assessed [12].

Given its broad scope of implementation, the interRAI CPS scale could potentially provide a ‘common language’ across settings and professions to enable individuals and services to be followed-up and compared across time in respect of their degree of cognitive impairment [13]. It could also potentially offer considerable utility in public health planning and research contexts without requiring additional tests outside of routine care [14].

The interRAI and the derived CPS were originally developed for use in aged residential care, but it is the CPS derived from community care instruments that is of most potential use to the health sector. The practical usefulness of the CPS in this role completely hinges on the validity of the CPS being derived from use of the interRAI Home Care Assessment tool (interRAI-HC) with older people in the community. The majority of validation studies have compared the CPS with other cognitive tests such as the cognitive screening instrument, the Mini Mental State Examination (MMSE) [10], reporting a moderate to strong correlation. Previous research has generally focused on aged residential care [14,15,16,17,18], but there have been some studies in acute hospital settings [12,13,19] and mental health care settings [20,21]. Although the interRAI-HC instrument is the most widely used of the non-residential interRAI versions, the evidence base for the use of the CPS in this setting is sparse. To our knowledge, only one study has focused on people receiving care in their own homes [22], with one further study collapsing data for a version of the CPS across a range of settings, including home care [23]. It is this gap that this study begins to address.

Furthermore, few studies have examined the discriminative validity of the CPS against a clinical diagnosis of dementia. Two exceptions are Pasquay et al. [24] in an aged residential care setting and Travers et al. [19] in an acute hospital setting. Of particular note, Travers et al. [19], found the CPS to have poor sensitivity in the acute hospital setting, and similar concerns have been raised by studies comparing the CPS with the MMSE cut-off in acute care settings [12,13]. It has been suggested that the more limited interactions between raters and clients in non-residential settings compared with those in aged care facilities could affect the performance of the CPS [12].

The aim of the present study is to explore the performance of the interRAI-HC CPS in a sample of older adults with known cognitive status. It is a pragmatic exploratory study rather than being a formal validation study. Whilst limited, this is one of the first studies that begins to develop our understanding of the strengths and weaknesses of the CPS in real world community settings.

## 2. Materials and Methods

### 2.1. Study Design and Participants

This is a retrospective diagnostic case series. The initial sampling frame was all individuals aged 65 or over who had been discharged from an acute psychogeriatric inpatient unit or a specialist memory clinic in two large New Zealand district health board areas during a set period. These services provide a specialist assessment environment, ensuring a high level of confidence in the accuracy of dementia diagnoses. The time frame was between 1 June 2013 and 31 May 2014 for the Canterbury District Health Board and between 1 June 2012 and 31 May 2014 for the Auckland District Health Board (because fewer interRAI assessments were available from that area at the time). Where there was more than one eligible episode of care for an individual within the specified time period, only the most recent episode was used.

The inclusion criteria were that:

(i) an interRAI Version 9.1 Home Care assessment was completed within 90 days of discharge from psychogeriatric inpatient care or memory clinic assessment;
(ii) participants gave permission, at the time of their interRAI assessment, for the interRAI data to be used for research purposes.

Of the 336 individuals discharged or with a completed memory clinic assessment from the Canterbury District Health Board services, 118 were administered an interRAI in the time frame, and of these, 97 gave permission for their data to be used (82%). Of the 276 relevant individuals from the Auckland District Health Board services, 43 were administered an interRAI in the time frame, and of these 37 gave permission for their data to be used (79%). The final sample included a total of 134 individuals.

### 2.2. Measures

#### 2.2.1. Index Test: Interrai Cognitive Performance Scale (2005 Revision)

The interRAI data were collected using version 9.1 of interRAI-HC in the context of standard care by clinically registered and certified interRAI assessors during a face-to-face structured assessment with the older adult and a significant other if required. Competency in interRAI assessment is achieved by attending a 3-day interRAI training programme, completing ten assessments and care plans, passing an evaluation and achieving an acceptable quality review outcome.

The CPS scores are determined by the algorithm shown in Figure 1 above, using items concerning daily decision-making ability, short term memory, procedural memory, the ability to make oneself understood and the ability to feed oneself, and whether the individual was in a coma.

#### 2.2.2. Reference Standard: ‘Clinical Dementia Diagnosis’ or ‘No Clinical Dementia Diagnosis’

In this study, the research question focuses on the potential practical application of the CPS as a tool to identify possible dementia. As such, the appropriate gold standard for comparison is a clinical diagnosis of dementia. It should be noted, however, that scores on the CPS can be impacted by a variety of diagnoses, for example, delirium, head injury, or mild cognitive impairment.

The clinical diagnosis of dementia was made by a geriatrician or old age psychiatrist, or a psychiatry resident under their supervision, in the context of a multidisciplinary assessment environment—either a memory clinic or an acute psychogeriatric AT&R (assessment, treatment and rehabilitation) ward. A recorded dementia diagnosis was coded if it was listed in the comprehensive problem list or detailed within the discharge summaries or letters for the episode. Where there was any lack of clarity, the medical records were reviewed by a consultant psychiatrist of old age (M.C.) to determine what the clinical team’s contemporaneous categorisation had been. 

### 2.3. Data Collection

This study was approved by the Health and Disability Ethics Committee and the interRAI New Zealand Governance Board. Patient characteristics and the presence or absence of a dementia diagnosis were collated from patient discharge summaries and medical records. The routinely collected interRAI data for the relevant sample were extracted from the New Zealand national data repository.

### 2.4. Data Analysis

Th analysis was conducted using the IBM SPSS for Windows, Version 27 (IBM, Armonk, NY, USA) and MedCalc for Windows, version 20 (MedCalc Software, Ostend, Belgium). The overall diagnostic accuracy of the CPS was assessed by the area under the receiving operating characteristic curve (AUC). In the present study, the AUC represents the probability that a randomly selected individual from the subgroup with a diagnosis will have a lower cognitive test result than a randomly selected individual from the subgroup without the diagnosis. An AUC between 0.9 and 1.0 was judged as indicating ‘excellent’ accuracy, 0.8 to 0.9 as ‘good’, 0.7 to 0.8 as ‘not good’, and 0.6 to 0.7 as ‘worthless’ [25]. As the cases were all purposively sampled, the receiving operating characteristics of the CPS are specific to the sample.

Diagnostic accuracy for the CPS for the clinical diagnosis of dementia using the recommended cut point (1/2) and using a higher cut point of 2/3 were calculated including:

- Sensitivity: probability that the CPS score will be ‘positive’ (over cut-off) when the diagnosis is present.
- Specificity: probability that the CPS score will be ‘negative’ (under cut-off) when the diagnosis is not present.
- Positive likelihood ratio: ratio between the probability of a positive (over cut-off) CPS score given the presence of the diagnosis and the probability of a positive (over cut-off) CPS score given the absence of the diagnosis, i.e., Sensitivity/(1-Specificity).
- Negative likelihood ratio: ratio between the probability of a negative (under cut-off) CPS score given the presence of the diagnosis and the probability of a negative (under cut-off) InterRAI scale score given the absence of the diagnosis, i.e., (1-Sensitivity)/Specificity.
- Positive predictive value: probability that the diagnosis is present when the CPS is ‘positive’ (over cut-off).
- Negative predictive value: probability that the diagnosis is not present when the CPS score is negative (below cut-off).

As a rule of thumb, sensitivity or specificity values of 0.8 or more were considered good, 0.7 to 0.79 fair, and less than 0.7 poor. A positive likelihood ratio of greater than 10 or a negative likelihood ratio of less than 0.1 was considered a large change in probability, a positive LR of 5 to 10 or negative LR of 0.1 to 0.2 moderate, a positive LR of 2 to 5 or negative LR of 0.5 to 1.0 small (but sometimes important), and a positive LR of 1 to 2 or negative LR of 0.5 to 1 small and rarely important [26]. 

## 3. Results

### 3.1. Sample

Across the 134 participants, the mean age was 78 years (range: 65 to 95 years) and 51% were female. The majority of participants (63%) were identified as New Zealand European, 5% as Māori (indigenous New Zealanders), 2% as Pacific Island people, 2% as Asian, and the remaining 30% were recorded as ‘unspecified’ or ‘other’ (including other European).

A dementia diagnosis was recorded to have been made in 54% of cases (*n* = 72), with Alzheimer’s and mixed dementia (both Alzheimer’s and vascular) being the predominant subtypes. Mild cognitive impairment (exclusive of dementia) was diagnosed in 11% of participants (*n* = 15). We included people with mild cognitive impairment in the ‘no clinical dementia diagnosis’ group because excluding them would increase the risk of ‘spectrum bias’ and result in spuriously accurate results [27].

### 3.2. Accuracy for Diagnosis

As can be seen in Table 1, the proportion of dementia diagnoses increased with increased CPS score. The overall accuracy of the CPS in predicting dementia diagnosis in this sample was good, with an area under the Receiver Operating Characteristic (ROC) curve of 0.82 (95% CI = 0.75–0.89). Table 2 summarises the performance of CPS using the cut points of 1/2 and 2/3. The optimal cut point was 1/2, coinciding with the recommended cut point. Using this cut point, the CPS showed good sensitivity (0.90) but poor specificity (0.60), with a small positive likelihood ratio. The Youden J statistic of 0.50 was on the borderline of acceptability for a diagnostic test.

Using an alternative more conservative cut point of 2/3, the positive predictive value improved to 0.89 with a moderate positive likelihood ratio.

## 4. Discussion

This is one of the first studies to examine the performance of the CPS against dementia diagnosis by specialist assessment. In our population of older adults recruited from specialist services, this study found the CPS at the recommended cut-off point of 1/2 to have good overall accuracy, with high sensitivity but poor specificity, when compared with dementia diagnosis from a specialist team. Using a higher cut point of 2/3, the positive predictive value improved, suggesting 89% of older adults in this sample with a CPS score of 3 or above truly had a diagnosis of dementia.

If replicable in representative community samples, the high sensitivity of the CPS in this study population may open the possibility for the CPS in the interRAI-HC to be as a screening tool to ‘red flag’ community dwelling older adults to be referred for a more comprehensive cognitive assessment. From a public health perspective, to have a cognitive screening tool available in a widely used routine assessment of vulnerable older adults changes the cost–benefit ratio of screening considerably. While there is controversy over the value of cognitive screening in the general population over the age of 65 [28], it is important to note that the interRAI population is not a general sample of the older population. The interRAI clients receive the assessment because of potential vulnerabilities that may meet the criteria for government funding support at home or in aged residential care. They typically have chronic diseases and/or functional impairment [29]. The interRAI relies on the reporting of noticed symptoms in a population with a relatively high prevalence of dementia. One in four individuals receiving the interRAI Home Care in New Zealand have a diagnosis of dementia [30], and another 1 in 20 may have signs of cognitive issues but no diagnosis [31]. The CPS might be expected to have lower sensitivity and specificity in the general interRAI-HC population compared with this study’s specialist service sample because the proportion of people with mild dementia and the proportion of people with non-dementia conditions that trigger the CPS questions is likely to be higher in that population.

There was a notable contrast in the properties of the CPS according to the cut point used. A threshold of 1/2 had high sensitivity but poor specificity. A threshold of 2/3 had poor sensitivity, but good specificity. This suggests a limitation of the CPS in this role, forcing a choice according to practical priorities according to purpose.

The cut-off of 1/2 provided the best trade off of sensitivity and specificity overall in this sample, with 76% accuracy. High sensitivity may provide security for situations in which an overestimate is acceptable—for example, if estimating an upper bound for the possible prevalence of cognitive impairment in this vulnerable group for planning purposes.

If the priority is to target individuals who are highly likely to have dementia for further assessment as a gateway to better support, then the 2/3 threshold may be more appropriate than the 1/2 threshold. Since over 30,000 interRAI-HC assessments are completed in New Zealand each year [32], the high false positive rate yielded by the 1/2 threshold would entail significant wastage of limited health resources and could also lead to many positive people being subjected to unnecessary worry from being diagnosed with dementia.

In this sample, 89% of people with a score of 3 or above were true cases of dementia. Assuming that future work confirms the generalisability of our findings, people who are scored 3 or above on the CPS who do not have a formal diagnosis of dementia could be rationally advised to undergo further cognitive assessment. This could represent a sizeable outreach: a previous study using interRAI found that a third of older New Zealanders with a CPS score of 3 or more did not have a diagnosis of dementia, even after excluding individuals with possibly confounding comorbidities such as depression and other neurological conditions [31], representing 1 in every 20 people receiving the interRAI. Of particular note in that study was the over-representation of people who were isolated, living alone, and Asian, amongst those with possible undiagnosed dementia. The interRAI CPS may help clarify whom to offer cognitive screening outreach to among those who are less likely to seek out help from their general practitioner. More research is needed following up an interRAI sample with possible undiagnosed dementia who are referred for further assessment to gauge the ratio of false positive to true positives, and the emotional and monetary costs and benefits.

Even with the higher than standard cut-off and relatively high baseline prevalence of dementia in this sample, the false positive rate for the threshold of 2/3 was still 11%. This provides a salient reminder that screening tests such as the CPS are never a substitute for a thorough assessment.

A different concern about screening for dementia that is sometimes, advanced centres doubt the value of an early diagnosis itself where a cure is not available [28], but this overly medical view misses the point. The value of the diagnosis is determined in a large part by the usefulness of the options and supports it can unlock. This is as true for dementia as it is for all chronic, incurable, non-communicable diseases. As the New Zealand Dementia Action plan notes: “People with dementia and their family/whānau care partners/supporters can enjoy more full, active and meaningful lives when they have access to a timely, accurate diagnosis as well as to the right support and assistance [33].”

New Zealand has never had a community dementia prevalence study using accepted modern methods. Between 10 and 40% of all New Zealanders aged 65 years and 85 years, respectively, have had an interRAI-HC [34]. If further research supports the present findings, the CPS used with a cut point of 2/3 could be considered as a proxy for dementia in estimating the prevalence of dementia in the interRAI population.

While the interRAI offers an avenue of screening with impressive outreach, questions still remain about the appropriateness of the interRAI-HC CPS as a screening tool. Our finding of high sensitivity/poor specificity is at odds with previous research from non-residential settings where poor sensitivity/high specificity was reported [12,13,19]. Comparison with these previous studies is tempered by the difference in setting and determination of dementia. For example, the studies by Bula and Wietlisbach [12] and Wellens et al. [13] used MMSE ≤ 23 as their proxy for the presence of cognitive impairment, while in the Travers et al. study [19] the diagnosis of dementia was made by two physicians independently reviewing medical records and assessments, without examining the participants. In the present study, all our participants were assessed in person by specialist services.

### Limitations

The specific sampling frame of the current study allowed us to be confident in the dementia diagnoses, but at the cost of limited generalisability. Our sample was drawn from people who had engagement with specialist psychogeriatric services or memory clinics; therefore, the rate of dementia, and other patient characteristics, will not be representative of the whole population assessed using the interRAI-HC. In particular, the engagement with these specialist services may have made the participants more aware of their cognitive problems if present, improving the quality of self-report.

As the interRAI assessments were collected as part of usual clinical care, interRAI assessors were not blinded to the participants’ diagnosis of dementia and would have had access to medical records at the time of assessment. However, interRAI assessors are not involved in calculating the CPS scores which are generated by a computerised algorithm after an interRAI assessment using six independent items (daily decision-making ability, short term memory, procedural memory, the ability to make oneself understood and the ability to feed oneself, and whether the individual was in a coma). In 2015, an update to the CPS was released, the CPS2 [23]; however, the CPS is still the version generally reported in New Zealand. Future research could explore any improvement in the validity of the CPS2 over the CPS.

## 5. Conclusions

The CPS is a very attractive cognitive measure as the interRAI is widely used in New Zealand, but further research is needed before the CPS is interpreted as a cognitive screening tool for ascertaining the diagnosis of dementia. If the performance of the interRAI CPS Home Care is confirmed in further studies, older adults with a CPS of 3 or above, but without a formal diagnosis of dementia, should be referred for further cognitive assessment given the high probability that they truly have dementia.

## Figures and Tables

**Table 1:** Clinical diagnosis of dementia and no dementia by CPS score (*n* = 134).

CPS Score	Clinical Diagnosis	
Description	No Dementia (N = 62)	Dementia (N = 72)
0. Intact (*n* = 23)	21 (91%)	2 (9%)
Borderline intact (*n* = 21)	16 (76%)	5 (24%)
2. Mild impairment (*n* = 54)	21 (39%)	33 (61%)
3. Moderate impairment (*n* = 25)	3 (12%)	22 (88%)
4. Moderately severe to very severe impairment (*n* = 11)	1 (9%)	10 (91%)

**Table 2:** Performance of the Cognitive Performance Scale using cut point of 1/2 and 2/3.

Cut Point	Sensitivity (95% CI)	Specificity (95% CI)	Positive Likelihood Ratio (95% CI)	Negative Likelihood Ratio (95% CI)	Positive Predictive Value (95% CI)	Negative Predictive Value (95% CI)	Accuracy (95% CI)
1/2	0.90 (0.81–0.96)	0.60 (0.46–0.72)	2.24 (1.64–3.06)	0.16 (0.08–0.34)	0.72 (0.66–0.78)	0.84 (0.72–0.92)	0.76 (0.68–0.83)
2/3	0.44 (0.33–0.57)	0.94 (0.84–0.98)	6.89 (2.58–18.40)	0.59 (0.48–0.74)	0.89 (0.75–0.96)	0.59 (0.54–0.64)	0.67 (0.59–0.75)

## Data Availability

The data presented in this study are available on request from the corresponding author. The data are not publicly available due to the requirements of the ethical approval given.
